# Reducing Inpatient Pediatric Central Line–associated Bloodstream Infections by Proactive Risk Assessment and Escalation

**DOI:** 10.1097/pq9.0000000000000928

**Published:** 2026-09-09

**Authors:** Chris I. Wong, Claudia Hoyen, Joyce Deptola, Theresa Smith, Josephine Rey, Charles G. Macias

**Affiliations:** From the * Division of Pediatric Hematology-Oncology, Department of Pediatrics, University Hospitals Rainbow Babies and Children’s Hospital, Cleveland, Ohio; † Division of Pediatric Hematology-Oncology, Department of Pediatrics, University Hospitals Rainbow Babies and Children’s Hospital, Cleveland, Ohio; ‡ Department of Pediatrics, University Hospitals Rainbow Babies and Children’s Hospital, Cleveland, Ohio; § Department of Pediatrics, University Hospitals Rainbow Babies and Children’s Hospital, Cleveland, Ohio.

## Abstract

**Introduction:**

Central line–associated bloodstream infections (CLABSIs), despite the application of best practices, are still prevalent and cause significant morbidity and mortality in pediatric patients. We describe a quality improvement project with an aim to reduce inpatient CLABSIs focused on standardization and proactive risk assessment.

**Methods:**

The aim was to reduce inpatient CLABSI rates by 30% within 3 years beginning in January 2021. An apparent cause analysis in 2018 suggested that the lack of escalation of concerns was a potential focus area. We used Plan-Do-Study-Act cycles starting with a pilot in 2019 to roll out a CLABSI Watcher Program. After a CLABSI prevention summit in 2020, a reorganization of drivers reidentified the need for a standardized escalation process for central line (CL) concerns. A CL nurse program was then scaled across the hospital, focusing on timely proactive huddling. Hospital-wide standardization of practices occurred from 2021 to 2023. We used statistical process control charts to assess changes in CLABSI rates.

**Results:**

Special cause variation, with a downward shift in the CLABSI rate process mean compared with baseline (0.97 per 1,000 CL days), was observed starting June 2021 (0.13 per 1,000 CL days). A second shift occurred from August 2023 through December 2024 (0.00 per 1,000 CL days). The CLABSI rate decreased by 65% (0.34 per 1,000 CL days) through December 2023.

**Conclusions:**

Significant reductions in hospital CLABSI rates can be achieved by using additional measures beyond focusing on CL maintenance and insertion bundles, and particularly through developing proactive risk assessment and escalation processes.

## INTRODUCTION

Central line–associated bloodstream infections (CLABSIs) are among the most common healthcare-acquired conditions in acute care hospitals. Although there have been substantial reductions in CLABSIs, a pediatric mortality rate of 50% has been documented.^1^ Beyond the negative patient safety impact, CLABSIs impose an institutional financial burden, with an estimated cost of $55,000 per infection.^2^ Using prevention bundles for insertion and maintenance of central lines (CLs) can reduce CLABSI rates if implemented and performed reliably.^3^ Solutions for Patient Safety (SPS), a network of children’s hospitals dedicated to eliminating healthcare-associated harm, has adapted these bundles for pediatric care.^4^

However, despite the successful implementation of bundles, many organizations still experience higher rates of CLABSIs than expected and encounter challenges in sustaining improvement work.^5^ Adherence to CLABSI prevention bundles requires knowledge of the bundle and engagement of the healthcare team.^6^ Along with engagement, team communication is essential for escalating and mitigating risks.^7^

A growing body of evidence suggests that traditional root cause analyses of individual safety events may not capture the broader systemic issues contributing to infection rates.^8^ Instead, common cause analysis (CCA)—examining patterns across multiple CLABSI events—has revealed valuable insights into recurring themes such as staffing challenges, inconsistent or ineffective supply chain practices, and communication breakdowns, as experienced during the COVID-19 pandemic.^9^ Principles of Safety I (reactive measures to prevent harm) and Safety II (proactive efforts to ensure safe outcomes) are increasingly recognized as essential components of effective CLABSI prevention and disaster preparedness, including during pandemics.^10,11^

In response to the surge of the COVID-19 global pandemic in 2020, our hospital developed new strategies to reduce inpatient CLABSI rates, despite the challenges the pandemic presented.

## METHODS

### Context

Rainbow Babies and Children’s Hospital is a pediatric acute care academic hospital in Cleveland, Ohio, and is part of the University Hospitals integrated network system. All patients admitted to any of the hospital’s 244 beds, including the neonatal intensive care unit (NICU) and pediatric intensive care unit, were included in the analysis.

### Definitions and Data Collection

Our SMART aim was to reduce and sustain our inpatient CLABSI rate by 30% within 3 years starting in January 2021. For this effort, the focus was exclusively on non-mucosal-barrier injury CLABSI events. Initially, CLABSI data were manually collected across all units. Following the implementation of a new electronic health record system, data collection transitioned electronically. A validation process was conducted for 4 months and completed by December 2023. Positive blood cultures in patients with CLs were reviewed by infection control (IC) specialists (infection preventionists) according to National Healthcare Safety Network definitions.

### Designing the Intervention: Current State Analysis

Our institution has had a long-standing multidisciplinary CLABSI prevention team as part of the hospital’s quality strategic priorities.^4^ An increase in the 2018 CLABSI rate (20 CLABSI events with a rate of 1.24 CLABSIs/1,000 CL days, compared with the 2017 rate of 0.83/1,000 CL days) prompted a retrospective review by the group to identify common themes. This increase occurred despite multiple months of reported adherence to our evidence-based CLABSI prevention maintenance and insertion bundles (92% and 99.7%, respectively). From this analysis, we found that 10 out of the 20 CLABSI-related apparent cause analyses identified issues (eg, dressings not intact, patient-specific skin issues, frequent dressing changes, CL integrity, and multiple CL entries) with effective, timely communication and escalation of concerns involving the CL within 72 hours before the date of the positive blood culture. As a result, some limited interventions (eg, reeducation on dressing applications to prevent unanticipated dressing changes, product changes due to CL tubing, and filter issues resulting in CL integrity breaks) were implemented in late 2019 and early 2020 to focus on the rapid escalation and resolution of concerns to prevent CLABSI.

In May 2019, a multidisciplinary CLABSI Watcher Program (CWP) was created by the hospital quality/safety leadership and the IC team (both including physician leaders). In addition to those representatives, it also included CL insertion and maintenance nurse experts (with a subcommittee called the divisional intravenous access committee driven by the increased use of peripherally inserted central catheter insertion team nurses), as well as designated CLABSI champions from different hospital units (including proceduralists inserting CLs). The CWP was developed for risk identification, mitigation strategies, and escalation of concerns with CLs, such as dressing integrity, line removal delays, and contamination risks. We developed the term “CLABSI watcher” as a flag in the medical record for patients with characteristics assessed as “high-risk” for CLABSI, based on any of these specific criteria, after adapting them from the criteria of an existing hospital.^12^ (**See Supplemental Digital Content 1**, which displays the CLABSI Watcher Tool, https://links.lww.com/PQ9/A820.) The CWP documented individualized plans in the patient’s chart by the IC physician leader in collaboration with frontline staff, including residents, faculty, and bedside nurses, to mitigate CLABSI risk. Patients with a “CLABSI watcher” designation received more frequent and detailed assessments of the CL, environment, and care provided by the primary team, with support from local CLABSI champions and the DIVA committee. A team member from the CWP was designated as the follow-up leader.

The CWP was piloted primarily in the NICU as one of our high-risk areas. The NICU first began piloting a project to implement nurse-driven daily discussions of the CL, with the eventual implementation of a formal NICU CL nurse role in January 2020.

Amidst the pressures of the COVID-19 pandemic, the leadership team held a CLABSI prevention summit in December 2020 to prioritize efforts and engage participation. Given high bundle adherence and rising rates of nurse CL competencies documented during reassessments, key drivers identified by the stakeholders focused on using Safety II principles while addressing the pressures imposed by the pandemic. Key drivers included engagement of local and hospital champions as leaders to influence change, reliability of leaders to influence change, adherence to the SPS CLABSI prevention maintenance bundle, and high-risk identification and mitigation processes. CLABSI data in 2020 demonstrated that 93% of patients identified as “CLABSI watchers” (n = 71) did not experience a CLABSI, and that 60% of CLABSI events (n = 20) occurred in patients who had not been identified as “CLABSI watchers.” This suggested that the mitigation strategies implemented by the CWP were successful, but that additional interventions would be needed to improve CLABSI rates.

### Interventions

After the CLABSI summit in December 2020, the intervention period started in January 2021 and continued through December 2023. Sustainability efforts were initiated in 2024 to embed these practices into routine operations. This work was guided by the Model for Improvement framework and used Plan-Do-Study-Act (PDSA) cycles to iteratively refine interventions. Table 1 summarizes the interventions performed between 2021 and 2024.

**Table 1.** Summary of CLABSI Prevention Interventions and Dates of Occurrence During the QI Initiative

Time Period	Intervention	Safety Principle Applied
Early 2021	Expansion of CWP across the entire hospital	Safety II
March 2021	CWP ownership shifts from the hospital to the unit level, with a CL nurse dedicated to high-risk areas	Safety II
2021	Standardization of products and processes: antiseptic wipes, intravenous fluid, CL guidelines	Safety II
Late 2021	Proactive huddles. Secure, text-based communication is used to promote real-time escalation of CLABSI risks	Safety II
2022–2023	Planned transition to a new electronic health record promotes proactive clinical decision support and reporting to support risk identification and data turnaround	Safety II
January 2023	Four huddles along with CCA process become standard to review CLABSI events	Safety I and II
May 2023	Standardized designated clean work surface for CL care	Safety II
2024	Formalized role of medical director for the CL team and CLABSI prevention	Safety I and II

#### Expansion of the CWP and Formalizing the Role of Dedicated CL Nurses

The CWP project was expanded in 2021. We began introducing the CWP program and its criteria for “CLABSI watcher” across the hospital to ensure all frontline staff were familiar with the program and the term, with a focus on including providers (trainees, advanced practice providers, and faculty), as bedside nurses were already engaged. Upon identification of a “CLABSI watcher,” the CWP convened a same-day meeting with the primary team at the bedside to review the issue, discuss mitigation, and develop a plan documented by the IC physician leader. Although maintenance bundle practices had been standardized per guidelines^4^ before this project, there was a lack of standardization regarding the process for addressing individualized needs. Examples of individualized plans included different options for dressing change processes, addressing chlorhexidine gluconate sensitivity, decreasing CL entries, and developing a plan for potential escalation of CL removal. Provider involvement in CLABSI reduction efforts, including daily bedside discussions, was inconsistent. Once the problem was resolved, the CWP team signed off on the case. If the issue recurred, the “CLABSI watcher” status would be reinstituted.

Starting in March 2021, ownership of the CWP shifted to the unit level with the addition of a CL nurse role to all high-risk areas: the NICU, the cardiothoracic intensive care unit, the pediatric intensive care unit, and the hematology/oncology ward. Through the program, dedicated nurses assessed lines daily, provided oversight of the daily CLABSI prevention bundle, and escalated issues for resolution. In 2024, the hospital formalized the role of a medical director for the CL team to oversee CLABSI prevention efforts, provide clinical assessments, and facilitate bedside huddles for patients requiring clinical interventions.

#### Escalating Concerns in Real-time and Refining Huddles

In addition to the existing CWP process of huddling and developing an individualized plan, a real-time escalation process to include the CLABSI leadership team through the CL nurses was added. In late 2021, the introduction of a secure, text-based communication system for the hospital streamlined the timely reporting and management of line issues by the CL nurse program. This system enabled real-time or near-time communication among interdisciplinary teams, ensuring timely identification and resolution of challenges.

In January 2023, we expanded our process for conducting huddles and CCA to review systemic factors contributing to CLABSI events. We developed 4 types of huddles as part of the prevention program. First, CL nurses would initiate a huddle for “CLABSI watchers” or for any event that could be identified as a potential risk for CLABSI (eg, a discussion before transfer from one unit to another with a CL nurse and a bedside nurse who was not as familiar with daily CL care). Second, upon detection of any positive blood culture in a patient with a CL, the CWP and primary team huddled to determine whether other patient factors or alternative diagnoses should be considered that might better ascertain the etiology of the bacteremia. Third, upon formal designation of a CLABSI according to the National Healthcare Safety Networks definitions, a formalized questionnaire was developed to abstract pertinent information from the medical record or obtain input from the primary team. The multidisciplinary group, including the primary team, discussed the findings as a group to identify potential root causes and develop future action plans. Fourth, aggregate information and trends were reviewed as CCAs, and practices were refined to address any gaps identified. Additionally, the CLABSI leadership team began weekly meetings to review events and discuss interventions and strategies.

#### Standardization of Practices

In 2021, after implementing a series of PDSA cycles in select units, the hospital transitioned to standardized antiseptic wipes for all CL touchpoints. We also standardized hospital-wide intravenous administration sets, add-on devices, and neutral pressure valve infection port products in 2021 to reduce the risk of contamination during CL setups and minimize the need for adjustments during patient transfers.

An evidence-based guideline for CL selection, insertion, and removal was developed with a multidisciplinary group in 2021. The guideline provided algorithms for optimal decision-making and reinforced best practices.

In May 2023, we standardized a designated clean work surface for CL care across the hospital. Upon transition to a new electronic health record in 2023, the team proactively worked to prioritize clinical decision support and reporting that allowed teams to identify risk factors and provide rapid data turnaround for PDSA improvement work.

The program also addressed systemic barriers, such as standardizing timelines for transitioning temporary lines to permanent lines and ensuring timely removal of CLs.

PDSA cycles were used to trial the interventions described earlier. For example, the rapid escalation process began as an informal process at the bedside in the NICU that transitioned to a formalized trigger that used the existing hospital’s secure chat system to gather an interdisciplinary team at the bedside through multiple iterations of who should be included and how. Similarly, the process for transitioning temporary lines to permanent lines or ensuring timely removals evolved from informal discussions at the bedside to developing standardized processes aligned with decision support tools.

### Study of the Interventions and Analysis

The outcome measure was the change in non-MBI CLABSI rate per 1,000 CL days. Statistical process control charts, specifically u-charts with standard upper and lower control limits, were used to assess CLABSI rate change following rules of special cause variation.^12^ We tracked data for 1 additional year (2024) following the focused project interventions to assess the sustainability of the work.

Process measures included CL days and adherence to insertion and maintenance bundles, obtained through SPS standard audits, tracked using p-charts.^4,13^ The balance measure was the number of CLs that were recommended for removal by the CWP that resulted in reinsertion to evaluate for potential unintended consequences.

This quality improvement (QI) initiative was reviewed and determined not to meet the criteria for human subjects research by the University Hospitals Rainbow Babies and Children’s Hospital Center institutional review board. The article was prepared using the SQuIRE 2.0 guidelines.^14^

## RESULTS

### Outcome Measure

**Fig. 1.**
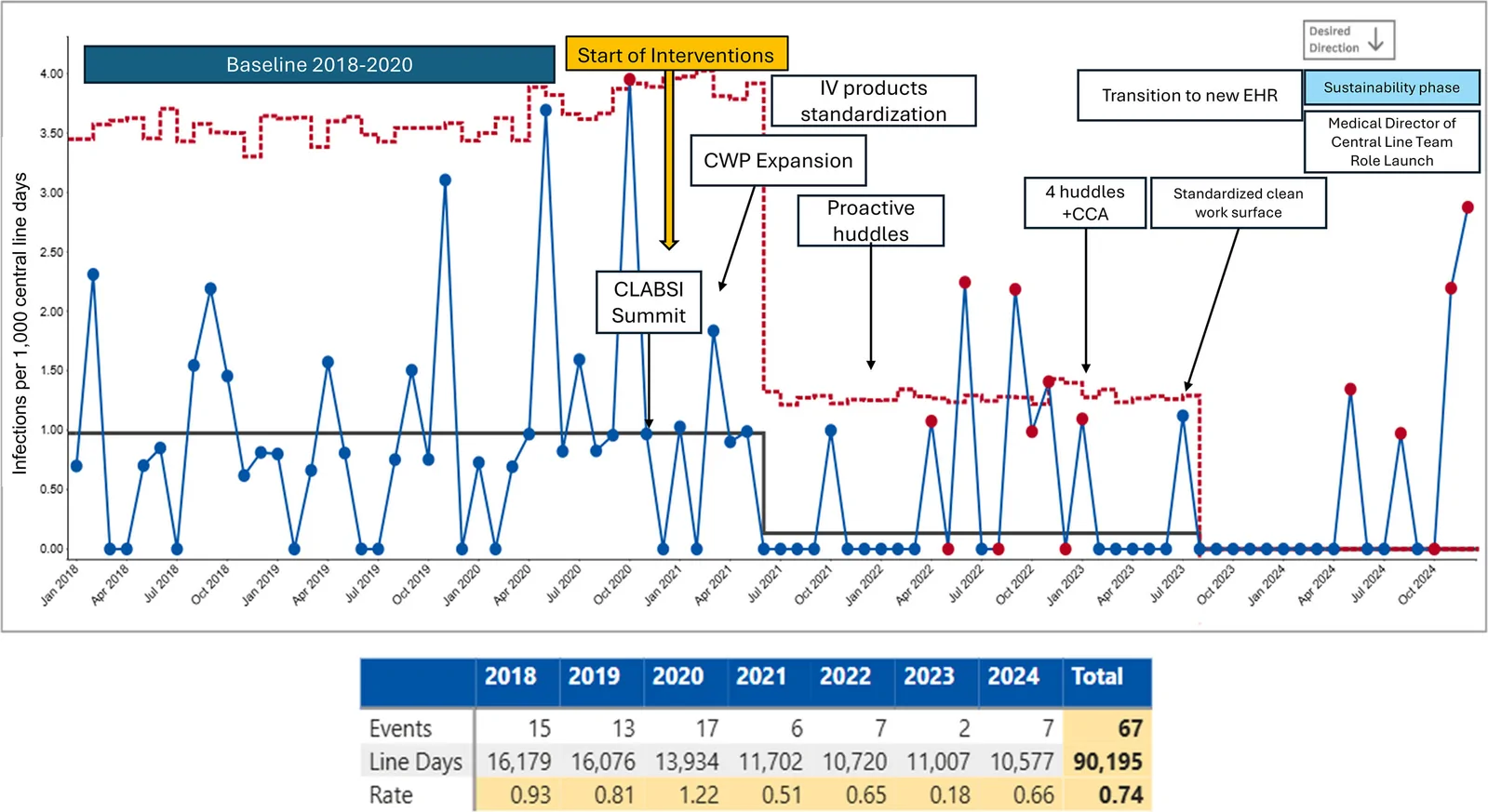
Statistical process control chart (u-chart, 3 sigma) demonstrating CLABSI rate and the change over time during the preintervention vs the intervention periods.

The baseline (2018–2020) CLABSI process mean (center line, 0.97 per 1,000 CL days) shifted downward to 0.13 (Fig. 1) starting in June 2021. A second downward shift in the mean occurred starting in August 2023 and continued through December 2024 (0.00 per 1,000 CL days), with 0 CLABSI events from August 2023 to April 2024. The CLABSI rate during the intervention period (2021–2023) was 0.34 per 1,000 CL days, a 65% reduction. The rate was 0.50 per 1,000 CL days from 2021 to 2024. The NICU experienced 1,545 days without a CLABSI, its longest (October 9, 2020–December 31, 2024) CLABSI-free period.

### Process Measures

**Fig. 2.**
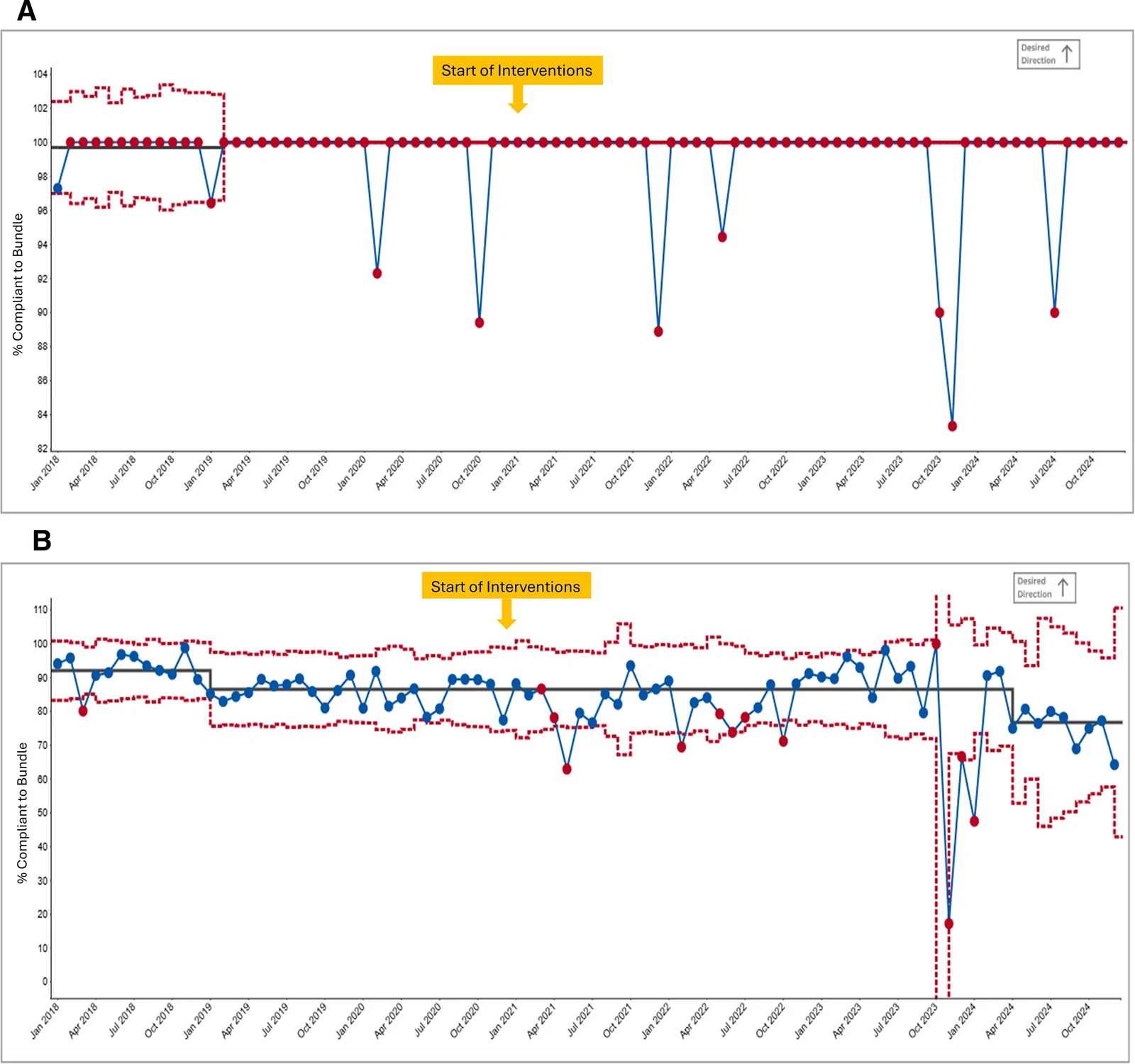
Statistical process control charts (u-chart, 3 sigma) demonstrating compliance with CL insertion (A) and maintenance (B) bundles.

The process mean for insertion bundle compliance (Fig. 2A) shifted upward (99%–100%) starting in February 2019. The maintenance bundle compliance (Fig. 2B) mean decreased from 92.1% to 86.6% starting in January 2019. In April 2024, the new downward-shifted mean was 76.8%.

The average number of CL days per year (2018–2020 versus 2021–2024) decreased from 15,396 to 11,142 (*P* = 0.02).

### Balancing Measure

No recommended CL removal by the CWP resulted in reinsertion during the same hospital encounter.

## DISCUSSION

Limited evidence exists regarding pediatric CLABSI prevention beyond bundle adherence, especially during resource constraints such as the COVID-19 pandemic. At our institution, rising CLABSI rates preceding the pandemic, along with the concern for a further anticipated rise due to the lack of dedicated time, effort, and resources once the pandemic began,^15–17^ presented an opportunity to use both Safety I and Safety II principles. CLABSI rate reduction has always been a hospital priority, but in 2019, the implementation of a new 5-year quality and safety strategy concentrated around the use of Safety I and Safety II principles. We surpassed our goal of a 30% reduction, achieving a 65% reduction in our CLABSI rate.

Although other pediatric institutions have used similar strategies in standardizing practices, bundle adherence, and risk mitigation to prevent pediatric CLABSI in certain populations,^12^ our work differs in the intentional approach to use Safety I and Safety II principles in response to an anticipated CLABSI rate increase, especially while attention was focused on mitigating the COVID-19 pandemic challenges. Despite a decrease in maintenance bundle adherence, we were able to decrease the CLABSI rate. Safety I principles, which focus on reactive measures, allowed us to drive standardization by using learnings from past events (apparent cause analyses) more holistically, complementing these with system-level CCA, focusing on specific high-risk situations rather than all situations, which was not possible due to a lack of resources. This translated to a proactive mitigation mindset (Safety II), supporting the development of the CWP and processes for escalating concerns, standardizing supplies, and improving resource allocation. As the attention shifted toward the emergent pandemic response, having dedicated resources for CLABSI prevention through CL nurses was only possible by identifying only the highest-risk patients requiring interventions rather than the entire hospital population.

Our work has limitations. QI work performed in a single institution can limit its generalizability. We believe the CWP was key to CLABSI reduction, although due to the nature of QI, it is difficult to attribute causality. We did not perform additional analyses to isolate the impact of individual initiatives, as this was intended as QI work. The decreased CLABSI rate in the setting of an expected decrease in maintenance bundle adherence during the postpandemic period suggests that the proactive work of the CWP contributed to this success. Our current state analysis suggested that patients with identified risks and mitigation plans were less likely to experience a CLABSI than those not identified as a “CLABSI Watcher.” We recognize that it is also possible that our stratification was incorrect, as we did not validate this risk model. Although there were no local changes beyond the challenges of the COVID-19 pandemic, our pediatric hospital began implementing a 5-year quality and safety strategy in 2019 and rolled it out concomitantly through the height of the pandemic starting in 2020. It is possible that, this contributed to our improved rates, as we interwove dedicated teams (quality coaches, analysts) and focused on a culture shift (adding Safety II principles) in all QI efforts. This work also required significant resources (eg, dedicated full-time equivalents), and therefore, it may not be possible to replicate these efforts in other settings. Nonetheless, our hospital’s NICU is a good representation of microsystem implementation as it piloted many of these initiatives, especially the CWP. Due to the staffing challenges observed during the pandemic, frontline staff served as champions in the NICU as the CWP nurses were shifted to direct patient care. Yet the NICU observed the longest period without a CLABSI, contributing to our hospital rate improvement. Concentrating efforts and resources in the highest-risk units may be a feasible alternative to reproducing this success elsewhere.

During the period without new interventions (sustainability), we observed an expected increase in the CLABSI rate, although it remained lower than baseline. During this time, there was less attention to CLABSI prevention, with greater focus on other strategic initiatives. Sustainability is challenging due to our dependence on human factors. Instead of concentrating on bundle adherence, we allocated our limited resources to developing a system that identifies the highest-risk patients and quickly escalates and resolves concerns. It is impossible to predict how long our efforts will be sustained, as they focus on promoting adaptive change within the culture. It is also difficult to discern if local trends in use during the pandemic (eg, more use of protective gear during CL care) could have impacted these results. Nonetheless, the impact of the COVID-19 pandemic was felt even during the intervention (eg, supply chain shortages), yet CLABSI rates still improved.

In conclusion, our QI initiative to reduce inpatient CLABSI focused on interventions beyond the bundle and proactive risk assessment, suggesting that this approach can lead to a reduction in inpatient CLABSI rates.

## ACKNOWLEDGMENTS

The authors thank the patients and their families for their partnership in this initiative. They also appreciate the time and sustained effort of the hospital staff, including the CWP and providers.

## Supplementary Material
