# Supplementary material for: Reducing Inpatient Pediatric Central Line–associated Bloodstream Infections by Proactive Risk Assessment and Escalation
Source: Pediatr Qual Saf. 2026 Sep 9;11(5):e928. doi: 10.1097/pq9.0000000000000928 (PMC13552822; doi:10.1097/pq9.0000000000000928)
Supplement: Supplementary file 1 [file pqs-11-e928-s001.docx]

University Hospitals
Rainbow Babies & Children's

# UH RBC CLABSI Watcher Tool

| Risk Category | Risk details | Possible Mitigation strategies | CLABSI Watcher Duration* |
|---|---|---|---|
| Contamination | • Soiled dressings or connection sites<br>• Contamination from stool or body fluid (other than blood)<br>• Unintentional disconnection or break<br>• Line in lower extremity (above knee) | • Position and dress catheter away from contamination source<br>• Utilize protective barrier/covering: e.g. Care-A-Line® or "mud-flap"<br>• Position tubing away from contamination source<br>• Utilize closed system medication line for intermittent med administration | 7 days<br>•Follow up every 72 hours or sooner if plan is not working |
| Line Integrity | • Breakage<br>• Tip migration<br>• Occlusion | • Change dressings if coiled/fragile part is out of dressing<br>• Use a securement device if lines are being pulled or have tension<br>• Position lines and tubing away from patient<br>• KVO all lines/lumens less than 2 Fr.<br>• Call PVAT or central line RN with any concerns about occlusions or Cathflo<br>• Follow Accidental Disconnection or Break algorithm for line, tubing, or cap break, cracks or disconnection<br>• Notify physician/APN of migration concerns; obtain X-ray; contact surgery/PVAT/neonatal PICC team or central line RN if needed | 7 days<br>• Follow up within 1st 24 hours and every 72 hours for breakage/tip migration<br>• Follow up every 48 hours for occlusion |
| Access | • Frequency of line entry for meds/IVF/labs<br>• Length of time CVC has been in place<br>• Multiple lumens | • Discuss line entries for possible consolidation/elimination during daily Collaborative rounds or central line rounds<br>• Discuss length of CVAD dwell time and alternate access or removal as Indicated by prescribed therapy/reason for line<br>• Discuss and consider ethanol or antibiotic locks if indicated<br>• Utilize closed system medication line for frequent med administration | 7 days<br>• Follow up within 24 hours for line not being utilized or alternate access indicated |
| Behavioral | • Picking<br>• Pulling<br>• Itching<br>• Developmental/parental concerns | • Reposition line away from patient access<br>• Re-educate pt./family utilizing key cards and other tools<br>• Utilize barriers for behavioral issue resulting in risk to line or Dressing- wraps/sleeves, similar items<br>• Use of distraction techniques/child life<br>• Ensure appropriate escalation to medical team<br>• Consult skin champions/ wound nurse if itching related to dressing and refer to CVAD dressing guide for further recommendations | 7 days<br>• Follow up within 1st 24 hours and every 48 hours after |
| Skin | • Compromised skin integrity<br>• Frequent dressing changes | • For skin breakdown, irritation, frequent dressing changes refer to CVAD Dressing Guide for alternate dressing strategies<br>• Consult wound nurse or dermatology for CHG allergy or dermatitis concern | 7 days<br>• Follow up within 1st 24 hours and at next dressing change. |
| Infection (or other concerns | • History of central line infection<br>• Infection concern of other site/source<br>• Immunosuppression ANC<500 | • Provide central line re-education in Family Learning Center for h/o CLABSI<br>• Clean high touch surfaces daily, provide 7 and 30 day room clean for long-term stay<br>• Review and engage patient/family in daily hygiene care<br>• Discuss ethanol/ABX locks or levofloxacin use in appropriate populations<br>• Consult infection control for other recommendations | 7 days<br>• Weekly until other infection risk has abated or ANC > 500 |

* At end of CLABSI Watcher duration, the CLABSI Watcher team or central line RN will re-evaluate need for continued follow up. If routine follow up is deemed not needed at that time, the CLABSI Watcher event note will indicate follow up as needed. The bedside team will notify CLABSI Watcher team or central line RN of additional risks or if previous mitigation plan needs adjusted or reviewed. If after the 7 day period, ongoing follow up is needed, a new 7 day period will begin with follow up as indicated for risk identified.

# NICU CLABSI Watcher Tool

Notify CLABSI Watcher Team with Risk Concerns

| | Risk Identification | Possible Mitigation Strategies | Duration Patient Remains a Watcher |
|---|---|---|---|
| C (Contamination) | ✓ Contaminated dressing or connection site<br>✓ Drainage under dressing<br>✓ Unintentional interruption of central line closed system | ✓ Position tubing up and away from contamination source<br>✓ Call Central Line RN<br>✓ Perform sterile Cap and/or Tubing change | ✓ 72 hours<br>✓ 72 hours<br>✓ 72 hours |
| L (Line Integrity) | ✓ Line Breakage<br>✓ Non-Central PICC line or low lying UVC<br>✓ Central Line requiring CATHFLO d/t occlusion | ✓ Clamp line as close to breakage and patient immediately, then call team<br>✓ Call CL RN<br>✓ Call CL RN | ✓ 72 hours<br>✓ Will remain watcher until able to obtain central access<br>✓ 72 hours |
| A (Access) | ✓ Frequency of line entry (6 X's or > in 24hr)<br>✓ Length of time line has been in place (Refer to reference guide)<br>✓ Line in lower extremity or groin (Refer to reference guide) | ✓ Consider consolidation, oral/ enteral meds if possible<br>✓ Discuss line day number each day in rounds<br>✓ Discuss line location each day in rounds | ✓ Until line is being accessed less (< 6X in 24 hours)<br>✓ Until line is removed<br>✓ Duration of line |
| B (Behavioral) | ✓ Developmental concerns (see reference guide) | ✓ Call Central Line RN | ✓ At discretion of the bedside RN, Central line RN, and Medical team |
| S (Skin) | ✓ Compromised skin integrity<br>✓ Frequent dressing changes (> than 1X/week) | ✓ Consult Central Line RN<br>✓ Consult Central Line RN<br>✓ Discuss concerns in rounds | ✓ Until skin issues resolve<br>✓ Until dressing remains on for > 7 days |
| I (Infection or other concerns) | ✓ Concern for infection at other site or source<br>✓ History of central line infection<br>✓ Immunosuppression | ✓ Clean Rooms<br>✓ CHG Baths | ✓ 72 hours post resolved issue<br>✓ Until patients is D/C'd<br>✓ Duration of line |
